# Social Determinants on Suicidal Thoughts among Young Adults

**DOI:** 10.3390/ijerph18168788

**Published:** 2021-08-20

**Authors:** Gang Wang, Liyun Wu

**Affiliations:** 1 School of Journalism and Communication, Wuhan University, 299 Ba Yi Road, Wuhan 430072, China; wangucb@whu.edu.cn; 2 School of Social Work, Norfolk State University, Brown Memorial Hall Suite 335.10, 700 Park Avenue, Norfolk, VA 23504, USA

**Keywords:** suicide, suicidal thoughts, poverty, religion, young adults, social determinants of health, national survey on drug use and health

## Abstract

Objective. The main objective of this study was to investigate the relationship between poverty, religion, and suicidal thoughts among U.S. youth. The disparities regarding gender, race, and ethnicity with regard to suicidal thoughts were also assessed. Methods. A cross-sectional correlational research design was used for this study and a national representative sample of 1945 young adults aged 18 to 25 was selected from the 2014 National Survey on Drug Use and Health. Logistic regression analysis with interaction effects was utilized to determine if poverty and religion were associated with suicidal thoughts. Results. About 43 percent of the sample reported having suicidal thoughts when things got worse and this prevalence rate varied by gender and race/ethnicity with white males self-disclosing the highest rate of suicidal thoughts. After adjusting for demographic and socioeconomic characteristics, black males who lived up to two times the poverty line had a higher likelihood of suicidal thoughts (*p* = 0.011), and religion protected against suicidal thoughts (*p* = 0.012). Youth with lower education and poor health were more inclined to have suicidal thoughts than their peers. Conclusions. Suicide is the second leading cause of death for American young adults aged 18 to 25. Understanding these differences between social determinants of suicide can help public health researchers strategize how to make evidence-based recommendations for suicide prevention efforts.

## 1. Introduction

Suicide is becoming a growing global public health concern in all regions of the world. Although precise global estimates of suicide rates are not easy to obtain, about 35 percent of World Health Organization (WHO) member nations have established comprehensive vital registration of suicides [1], which is defined as death caused by self-directed injurious behavior with intent to die as a result of the behavior [2]. The most recent statistics report that there are more than 700,000 people who take their own lives each year worldwide and the number of people who attempt suicide is much higher. By the income distribution, 23 percent of global suicides occur in high-income countries and 77 percent in low- and middle-income countries [3].

Suicide is the tenth leading cause of death in the United States. The Centers for Disease Control and Prevention [4] reported more than 47,500 deaths from suicide in 2019, which means that approximately 130 Americans die by suicide each day. Additionally, nearly 12 million adults seriously thought about suicide, 3.5 million planned a suicide attempt, and 1.4 million attempted suicide in 2019. Another data brief on suicide trends during the past twenty years from 1999 through 2019 by the CDC [5] disclosed that the age-adjusted suicide rate increased from 10.5 per 100,000 standard population in 1999 to 14.2 in 2018 and 13.9 in 2019. Over the same period (1999–2019), suicide rates were consistently higher for males compared with females. For instance, the male suicide rate was 22.4 per 100,000 population in 2019 versus the female suicide rate of 6.0 in 2019. As reported, the leading methods of suicide were among firearms, suffocation, and poisoning. All these data were extracted from the U.S. National Vital Statistics System mortality files.

Rates of suicide differ not only by gender but also by age group. Although suicide occurs throughout the lifespan, it affects individuals in various age groups differently and some age groups have higher suicide rates than others. Suicide has become the second leading cause of death for young people in the age range of 10 to 34, the fourth leading cause among people who are aged 34 to 54, and the fifth leading cause among people who are aged 45 to 54 [4]. People over the age of 65 represent 20 percent of the nation’s suicides, the highest suicide rate in the United States [6]. For instance, the suicide rates were higher for middle-aged adults from 45 to 54 (19.60 per 100,000) and adults from 55 to 64 (19.41 per 100,000), compared to adults 25 to 34 (17.54 per 100,000) and adults 35 to 44 (18.06 per 100,000). Among the senior populations who are eligible for social security: the oldest-old, who are 85 years or older, had the highest suicide rate (20.12 per 100,000); followed by middle-old adults, between the ages of 75 and 84 years, (18.64 per 100,000); and youngest-old, adults between the ages of 65 and 74 years (15.46 per 100,000). Young adults between the ages 15 and 24 years had the lowest suicide rate (13.95 per 100,000). Conejero and colleagues (2018) conducted a literature review to identify common suicide risk factors among the elderly population and their research findings highlighted factors such as feelings of social disconnectedness, neurocognitive impairment or decision making, chronic physical illnesses, and disabilities [7].

Suicide rates also differ substantially when looking into the combination of gender, race, and Hispanic origin. Overall, the non-Hispanic white population have higher suicide rates than the non-Hispanic black population and males, regardless of race and ethnicity, had much higher suicide rates than females [8]. The death rate from suicide for non-Hispanic white men (28.6 per 100,000) was 3.5 times greater than for non-Hispanic white females (8.0 per 100,000) in 2018. In comparison, suicide rates for non-Hispanic black men (12.2 per 100,000) were four times greater than for non-Hispanic black females (2.9 per 100,000). These disparities are connected to opposing factors which may either contribute to a higher suicide risk such as abuse, bullying, financial crisis, sexual violence, or community violence; or factors that may decrease the risk of suicide such as social support from family and friends, religious participation, resources and services from the community, easy access to healthcare, and other preventive programs [9,10,11,12,13,14,15]. Given the fact that suicide has long-lasting distressing effects on families, communities, and societies, it is imperative to understand the causes and determinants of suicide. According to the Office of Disease Prevention and Health Promotion [16], social determinants of health refers to “conditions in the environment in which people are born, live, learn, work, play, worship, and age that affect a wide range of health, functioning, and quality-of-life outcomes and risks”. Research findings based on young adult samples have provided empirical evidence about the social determinants of factors (low socioeconomic status, unequal distribution of resources, disadvantaged neighborhood) and subsequent inequalities in suicide rates [9,10,15].

The primary aims of this study are twofold: (1) to examine the association between religious attendance and suicidal thoughts among young adults; and (2) to determine the association between poverty and suicidal thoughts, controlling for major demographic and socioeconomic characteristics.

## 2. Literature Review

### 2.1. Suicide and Youth

Data from different sources have echoed the same findings that suicide is the second leading cause of death among young adults ages 18–25 [4], next to the leading cause of injury [17]. As its own developmental period, the period from ages 18–25 is sometimes referred to as “emerging adulthood”, “the frontier of adulthood”, or “the novice phase” [18]. From the lifespan perspective, this young adulthood signifies an important life stage transiting from children or adolescents to adults who can fulfill cultural expectations such as becoming financially independent and starting their own families. Youth in this age group experience many transitions, including graduating from high school, leaving home for college, facing school difficulties, looking for jobs, starting careers, forming new intimate relationships, making new friends, developing their own identity, moving from place to place for opportunities, changing living circumstances, and having family turmoil, to name but a few. With high expectations at the societal level, young adults may encounter stressful situations that trigger mental health problems such as anxiety, anger, confusion, depression, disappointment, hopelessness, insecurity, isolation, loss, stress, sadness, and withdrawal [19,20,21]. All these mental health challenges are associated with an increased risk of suicide and suicidal behaviors. Protective factors such as financial resources, access to health care, social support from families and friends, and effective communication can reduce the likelihood of suicide. In contrast, risk factors such as a depletion of financial resources, lack of health care, unsuccessful coping strategies, social isolation, and failure at conflict resolution may accelerate negative consequences such as suicide and suicidal behaviors.

### 2.2. Social Determinants and Suicide

Many social, economic, and environmental factors, otherwise known as social determinants of health, can potentially intensify or reduce the risk of suicide and suicidal behaviors. Understanding these differences across the course of life can help public health researchers strategize how to make evidence-based recommendations on suicide prevention efforts and contribute to ongoing policies with regard to youth development. Research findings in the literature suggest that suicidal behaviors are associated with many individual-level factors, including income, employment status, religion, mental health problems, personality characteristics, family factors, special life events, contagion and imitation, availability of means such as firearms and medications, previous suicide attempts, etc. [9,22,23]. Poverty, as an important social determinant of health, has been consistently proven to have a detrimental effect on health and developmental outcomes [24,25,26].

Furthermore, understanding the relationship between religion and suicidal behavior can help stakeholders manage, design, and implement effective prevention and intervention programs. An abundance of empirical studies have found that people with religious affiliation had less suicidal thoughts than unaffiliated people [9,27,28]. In terms of religious attendance, a study of American young college students found that attendance at religious services was associated with less suicidal ideation [29]. The same results emerged from a study of 36,984 Canadian adults and religious service attendance was related to a reduction in suicidal ideation [30].

In addition to personal factors, environmental factors such as school poverty and neighborhood poverty were also recognized as risk factors associated with mental health problems such as suicide [9,10]. A longitudinal survey of American adolescents from the National Longitudinal Survey of Adolescent Health across 132 middle and high schools found that boys were at a higher risk of attempted suicide than girls and the prevalence of attempted suicide and suicidal thoughts are both higher in impoverished schools compared to middle-income schools [10]. Another study of 2776 older adolescents from Canadian National Longitudinal Survey of Children and Youth also summarized that suicidal thoughts and attempts were significantly associated with neighborhood poverty after controlling individual characteristics [9]. Furthermore, the growing literature demonstrated that there were interaction effects between personal and environmental factors, suggesting that individual risk factors such as poverty are associated with enlarged effects in disadvantaged neighborhoods [31,32,33].

## 3. Methods

### 3.1. Data

This study utilizes the 2014 National Survey on Drug Use and Health (NSDUH), which was administered by the Substance Abuse and Mental Health Services Administration (SAMHSA) of the U.S. Department of Health and Human Services. The dataset is accessible for public use from the University of Michigan Inter-University Consortium for Political and Social Research (ICPSR) website (ICPSR ID 36361). The NSDUH measures the prevalence and correlates of drug use and mental health in the United States.

### 3.2. Sample Participants

The inclusion criteria for this study set limits on gender, age, and race/ethnicity. Both male and female respondents are included. The age range is from 18 to 25 years old. The racial/ethnic information includes three groups: white, black, and Hispanic respondents. With these restrictions in the sample selection, the final sample size includes 1945 participants.

### 3.3. Measurements

The demographic and socioeconomic variables include age, gender, race, marital status, employment status, and poverty status. Age is measured as a continuous variable with a numeric value. Gender is dichotomous with 1 = male and 2 = female. Race/ethnicity has three categories with 1= white, 2 = black, and 3 = Hispanic. Marital status is nominal with three categories: married, never married, and divorced or separated. Total family income is classified by poverty status: 1 = in poverty, 2 = up to two times the federal poverty line, and 3 = greater than two times the poverty line. Respondents’ overall physical health is measured in five categories: 1 = excellent, 2 = very good, 3 = good, 4 = fair, and 5 = poor. There are two indicators related to education. Respondents’ highest educational attainment is measured as an ordinal variable with four categories: less than high school, high school, junior college, and college and above. A binary variable is used to indicate whether the respondent is currently enrolled in school or not. The presence of suicidal thoughts was based on the survey question, “having suicidal thoughts when problems get worse”, and coded as binary with 1 = yes and 0 = no. The frequency of attending religious services in the past 12 months was an ordinal variable with 1 = zero times, 2 = 1 to 2 times, 3 = 3 to 5 times, 4 = 6 to 24 times, 5 = 25 to 52 times, and 6 = more than 52 times.

### 3.4. Hypothesis Testing

This study tested two hypotheses. Hypothesis 1 (H1) was to test whether more frequent religious attendance was associated with a lower likelihood of endorsing suicidal thoughts among young adults. Hypothesis 2 (H2) was to test whether living in poverty was associated with a higher likelihood of reporting suicidal thoughts among young adults. The bivariate Spearman correlation coefficients were presented to test the bivariate relationships between suicidal thoughts, religious attendance, poverty status, and all other demographic and socioeconomic variables. Logistic regression analysis was performed to test these associations along with odds ratios and 95% confidence intervals. Both hypothesis tests were two-tailed and the level of significance of 0.05 was utilized for statistical significance. All the statistical analyses were completed using SPSS version 26.0, IBM North America, New York, NY, USA.

## 4. Results

Respondents report their ideation pertinent to suicide. There were 837 respondents who had suicidal thoughts when problems get worse (43%) and 1108 respondents not having suicidal thoughts. Respondents indicated different frequencies of attending religious services in the past twelve months: ranging from the highest frequency with 912 of them never attending (47%), to the lowest frequency with 140 of them attending more than 52 times (7.2%).

The demographic and socioeconomic characteristics are presented in Table 1. The sample participants’ ages follow a pattern of even distribution with each age accounting for 12–13% of the total sample size. The sample participants’ age is distributed as follows: 242 of them were 18 years old (12.4%), 246 of them were 19 years old (12.6%), 235 of them were 20 years old (12.1%), 250 of them were 21 years old (12.9%), 492 of them were in the age range of 22 to 23 years old (25.3%), and 480 of them were in the age range of 24 to 25 years old (24.7%). There were more females than males: 1137 females (61.6%) and 708 males (38.4%). By combining gender and race/ethnicity, there are 512 white males (26.3%), 58 black males (3%), 138 Hispanic males (7.1%), 862 white females (44.3%), 140 black females (7.2%), and 235 Hispanic females (12.1%). The marital status is distributed as married (9.7%), never married (88.3%), divorced or separated (2.1%). In terms of total family income, which is measured by poverty status according to the federal poverty line, one quarter of the sample participants live in poverty (27.8%), another one quarter of the sample live up to two times federal poverty line (25.2%), and half of the sample live greater than two times the poverty line (46.9%). About fifty percent of the sample are currently enrolled in school (46.3%) and the other half are not. In terms of educational attainment, respondents having some college education account for the highest percentage (39.4%) and those less than high school degrees account for the lowest percentage (12.5%). 

Table 2 reports bivariate Spearman correlations between study variables. As displayed in Table 2, the presence of suicidal thoughts is negatively and statistically significantly associated with frequency of religious service (*p* < 0.01). In terms of gender and racial/ethnic disparities, white males are positively associated with the presence of suicidal thoughts (*p* < 0.05), and black females and Hispanic females are negatively associated with the presence of suicidal thoughts (*p* < 0.05). The physical health condition is statistically significant and being in poor health is positively associated with the presence of suicidal thoughts (*p* < 0.01). Black females are positively associated with living in poverty (*p* < 0.05), and so are Hispanic females (*p* < 0.01).

Table 3 displays the association between suicidal thoughts, religious attendance, poverty status, and other demographic and socioeconomic characteristics. There is a strong negative association between religious attendance and suicidal thoughts among this group of young adults (*p* = 0.012). As youth increase their frequency of religious attendance, they are less likely to come up with suicidal thoughts when things get worse. There are gender and racial disparities in terms of suicidal thoughts. Compared to female Hispanic participants, white males are more likely to consider suicidal thoughts when things get worse (*p* = 0.003). Similar patterns exist among black males and white females although they are not statistically significant. Despite the fact that black males as a whole are not associated with a likelihood of suicidal thoughts, differences in exposure to poverty plays an important role. The interaction terms between black male and living in up to 2X the poverty line indicators show that black males are highly likely to have suicidal thoughts compared to other racial/ethnic subgroups (*p* = 0.011). With regard to socioeconomic characteristics, educational attainment and physical health are both strongly associated with suicidal thoughts. Compared to young adults who obtain college or above degrees, participants with some college education indicate higher likelihood of have suicidal thoughts (*p* = 0.026). In comparison to participants with excellent, very good, or good physical health, young adults with fair or poor physical health are more likely to have suicidal thoughts (*p* = 0.025).

## 5. Discussion

Using the 2014 National Survey on Drug Use and Health, this study examined the relationships between religious attendance, poverty, and suicidal thoughts among different gender and race/ethnicity subgroups in the age range of 18 to 25 years old. About 43 percent of the sample reported having suicidal thoughts when things got worse and this prevalence rate varied by gender and race/ethnicity. Compared to other subgroups, white males had the highest prevalence of suicidal thoughts. Although black males as a whole did not exhibit a high prevalence rate of suicidal thoughts, black males who lived up to 2X the poverty line had a higher likelihood of having suicidal thoughts (*p* = 0.011) in comparison with black males living in other income brackets. In sharp contrast, religious attendance was negatively associated with suicidal thoughts in that higher frequency of religious attendance became a significant predictor of reducing suicidal thoughts (*p* = 0.012). In terms of other socioeconomic characteristics, educational attainment and physical health were both significantly associated with suicidal thoughts. Participants who had some college education indicated higher likelihood of having suicidal thoughts compared to those who obtained college or above degrees (*p* = 0.026). In addition, young adults with fair or poor physical health were more likely to harbor suicidal thoughts (*p* = 0.025) compared to their counterparts who had excellent, very good, or good health.

Participating in religion plays an important role in promoting mental health and reducing suicidal thoughts. While the literature has reported mixed empirical evidence of the relationship between religion and suicide, this complexity may be caused by the inconsistent conceptualization and operationalization of religious constructs. Religion has been measured in multiple ways; religious affiliation, religious participation or attendance, and religious doctrine. Different dimensions of religion may associate with different suicidal behaviors. However, religious attendance turned out to be a strong protective factor against suicidal behaviors [27,28,34].

Our findings also provide evidence that inequality of suicidal behavior is more common among young adults with lower family income and educational attainment. Poverty continues to be a stressor in life. In particular, youth living in impoverished communities may encounter more stressors than their peers living in middle-class neighborhoods. The study by Hoffmann and colleagues [35] analyzed a sample of 20,982 US youths aged 5 to 19 years old who committed suicide from 2007 to 2016 and identified an association between pediatric suicide and county-level poverty rate in the United States. With the increase in the county poverty concentration rate as defined by the percentage of the county population living below the federal poverty level, there is an increase in the likelihood for young people to die by suicide. The quantifying results compared US counties with poverty rates of at least 20% versus counties with poverty rates of less than 5% and identified that youth in high poverty counties were 37% more likely to die by suicide than their peers in low poverty counties. This interaction between personal and environmental factors magnified the negative effect of poverty on mental health problems. The research findings from this study also add empirical evidence related to the social determinants of health, one of the major objectives set up by *Health People 2030*, the 10-year national health agenda [36].

Within the public health sphere, there is a need for suicide prevention and intervention at multiple levels involving a wide array of sectors and stakeholders. Effective prevention and intervention programs can be established in school, community, and healthcare settings to address risk factors for suicide and save the lives of high-risk individuals. For instance, a multi-center, randomized controlled trial named Saving and Empowering Young Lives (SEYLE) in Europe recruited more than 11,000 school children in 168 schools from ten European Union countries and implemented three types of interventions to prevent suicide ideation or attempt and an evaluation of the program’s effectiveness was conducted with a 12-month follow up [37]. These schools were randomly assigned into three programs: a program training teachers and school personnel called Question, Persuade, and Refer (QPR), a program targeting students called Youth Aware of Mental Health Program (YAM), and a program focusing on screening and referral of at-risk students called Screening by Professionals (ProfScreen). The results reported a high efficacy of this school-based preventive intervention of suicidal behaviors by reducing suicidal attempts and suicidal ideation by approximately 50% based on the 12-month follow up data. Future research is needed to explore the mechanisms under which participants change their thought processes and adjust their behaviors.

We caution that our preliminary findings are based on cross-sectional data, which prohibited us from making causal inferences. It should be borne in mind that this relationship analysis did not resolve the common causality problems associated with observable data, including endogeneity and measurement errors. Despite these shortcomings in the research methodology, our findings advance the literature by focusing on the gender, racial, and ethnic disparities in suicidal thoughts using a US national representative sample. These findings provide insights into efforts to reduce suicide and suicide attempts and create family, school, and community support systems. The wide geographic coverage made the sample participants representative of different social, economic, and behavioral characteristics, and also had implications for other segments of populations in the world. Although this study is based on a US national sample, the association between poverty and suicide is consistent with findings based on low- and middle-income countries [38]. The research findings also concurred with a study from the 2001 Canadian National Longitudinal Survey of Children and Youth (NLSCY) which reported a significant association between neighborhood poverty and suicidal thoughts and attempts from a subset of 2776 sample participants [9].

## 6. Conclusions

Suicide and suicidal behavior have become growing public health problems around the world. This research sought to advance our understanding of how poverty disproportionately influenced certain gender and racial groups and how religion served as a buffer for reducing suicidal thoughts. The study findings offer important implications for health promotion. First, effective poverty reduction policies need to be in place to alleviate the income inequality in society and reduce vulnerabilities concentrated in disadvantaged schools and neighborhoods. The COVID-19 pandemic has hit the world unprecedentedly with 4 million deaths and 188 million individuals with a confirmed diagnosis [39]. In the United States, the pandemic caused 33.8 million confirmed cases and 606,190 deaths [40]. With the advent of the American Rescue Plan in 2021, one of the largest anti-poverty initiatives, thirty-five million American working families started receiving the first child tax credit payment of $300 or $250 starting 15 July 2021 [41]. Undoubtedly, this credit will help reduce the enormous social and economic losses caused by COVID-19 and increase the wellbeing of the general population. Second, many empirical studies of poverty and suicide are conducted within a deficit-factor paradigm. Our study sheds lights on a strength-based paradigm by emphasizing the role of religious attendance to promote young people’s mental and behavioral health. Future research exploring the mechanisms underlying these associations between poverty, religion, and suicide may foster a development of more effective suicide prevention and intervention programs and activities. Clinical treatments of mental health problems can link families to resources and services with regard to financial stability and coordinate care with community partners such as churches and other faith-based organizations.

## Figures and Tables

**Table 1:** Descriptive statistics of all variables, 2014 National Survey on Drug Use and Health (*n* = 1945).

Variables	Frequency	Percentage
Having suicidal thoughts when problems get worse		
Yes	837	43%
No	1108	57%
Frequency of attending religious services past 12 months		
Zero times	912	47%
1 to 2 times	256	13.2%
3 to 5 times	223	11.5%
6 to 24 times	250	12.9%
25 to 52 times	160	8.2%
More than 52 times	140	7.2%
Demographic Characteristics		
Age		
18 years old	242	12.4%
19 years old	246	12.6%
20 years old	235	12.1%
21 years old	250	12.9%
22 or 23 years old	492	25.3%
24 or 25 years old	480	
Gender x Race/Ethnicity		
Male, White	512	26.3%
Male, Black	58	3%
Male, Hispanic	138	7.1%
Female, White	862	44.3%
Female, Black	140	7.2%
Female, Hispanic	235	12.1%
Socioeconomic Characteristics		
Poverty status		
Live in poverty	520	27.8%
Up to 2X federal poverty line	471	25.2%
Greater than 2X poverty line	877	46.9%
Overall physical health		
Excellent	355	18.3%
Very good	818	42.1%
Good	556	28.6%
Fair	189	9.7%
Poor	27	1.4%
Marital status		
Never married	1717	88.3%
Married	188	9.7%
Divorced or separated	40	2.1%
Education attainment		
Less than high school	244	12.5%
High school	651	33.5%
Some college	766	39.4%
College and above	284	14.6%
Currently enrolled in school		
Yes	900	46.3%
No	1045	53.7%

**Table 2:** Bivariate Spearman Correlation for Study Variables, 2014 NSDUH (*n* = 1945).

	1	2	3	4	5	6	7	8	9	10	11	12	13	14	15	16	17
1. Suicide thoughts	1.000	-															
2. Religious service	−0.064 **	1.000	2212														
3. Age	−0.035	−0.069 **	1.000	-													
4. White male	0.063 *	−0.097 **	0.063 **	1.000	-												
5. Black male	0.012	0.067 **	−0.041	−0.105 **	1.000	-											
6. Hispanic male	0.015	−0.003	−0.003	−0.165 **	−0.048 *	1.000	-										
7. White female	−0.012	−0.024	0.020	−0.533 **	−0.156 **	−0.247 **	1.000	-									
8. Black female	−0.049 *	0.095 **	−0.008	−0.166 **	−0.049 *	−0.077 **	−0.248 **	1.000	-								
9. Hispanic female	−0.045 *	0.058 *	−0.084 **	−0.222 **	−0.065 **	−0.102 **	−0.331 **	−0.103 **	1.000	-							
10. Live in poverty	−0.006	−0.020	−0.065 **	−0.140 **	0.012	0.001	0.007	0.113 **	0.082 **	1.000	-						
11. Live below 2X poverty line	0.041	−0.009	0.058 *	−0.011	−0.006	0.008	−0.026	−0.006	0.055 *	−0.361 **	1.000	-					
12. Poor health	0.060 **	−0.060 **	0.039	−0.040	0.044	0.004	0.001	−0.016	0.040	0.064 **	0.006	1.000	-				
13. Never married	0.017	−0.060 **	−0.223 **	0.044	0.045 *	0.051 *	−0.080 **	0.058 *	−0.046 *	0.035	−0.092 **	0.002	1.000	-			
14. Less than high school	0.044	−0.051 *	−207 **	0.010	0.034	0.065 **	−0.054 *	−0.021	0.017	0.114 **	−0.028	0.079 **	−0.016	1.000	-		
15. High school	−0.014	−0.058 **	−0.162 **	0.007	−0.003	0.033	−0.054 *	0.013	0.038	0.034	0.036	0.048 *	−0.033	−0.269 **	1.000	-	
16. Some college	0.026	0.090 **	0.039	−0.016	0.001	−0.022	0.014	0.012	0.008	−0.051 *	0.015	−0.054 *	0.009	−0.305 **	−0.572 **	1.000	-
17. Not enrolled in school	0.015	−0.103 **	0.356 **	0.047 *	−0.025	−0.001	0.014	−0.025	−0.051 *	0.020	0.032	0.092	−0.200 **	−0.013	0.247	−0.301 **	1.000

** Correlation is significant at the 0.01 level (2-tailed) * Correlation is significant at the 0.05 level (2-tailed).

**Table 3:** Logistic regression model of suicidal thoughts, 2014 NSDUH (*n* = 1945).

Variables	Odds Ratio (95% CI)	*p*-Value
Independent variable		
Religious attendance	0.929 (0.877 to 0.984)	**0.012**
Gender x Race/Ethnicity		
Male, White	1.644 (1.178 to 2.293)	**0.003**
Male, Black	0.936 (0.381 to 2.302)	0.886
Male, Hispanic	1.400 (0.903 to 2.170)	0.132
Female, White	1.336 (0.982 to 1.817)	0.065
Female, Black	0.731 (0.332 to 1.608)	0.436
Female, Hispanic (reference)		
Poverty status		
Live in poverty	1.065 (0.835 to 1.357)	0.612
Live up to 2X poverty line	1.192 (0.936 to 1.518)	0.155
Greater than 2X poverty line (reference)		
Interaction terms		
Black male living in poverty	1.113 (0.298 to 4.147)	0.874
Black male living in up to 2X poverty line	9.631 (1.671 to 55.531)	**0.011**
Black female living in poverty	1.335 (0.527 to 3.381)	0.542
Black female living in up to 2X poverty line	1.429 (0.499 to 4.090)	0.506
Other demographic & socioeconomic variables		
Age	0.945 (0.883 to 1.011)	0.101
Current school enrollment status		
Not in school	1.173 (0.942 to 1.460)	0.155
In school (reference)		
Educational attainment		
Less than high school	1.417 (0.949 to 2.116)	0.088
High school	1.113 (0.801 to 1.547)	0.522
Some college	1.412 (1.043 to 1.911)	**0.026**
College and above (reference)		
Marital status		
Never married	1.102 (0.812 to 1.495)	0.534
Married or divorced (reference)		
Physical health status		
Poor or fair health	1.401 (1.043 to 1.882)	**0.025**
Excellent, very good, good (reference)		
Constant	0.725	0.503

## Data Availability

This study utilized public use data.
